# Integrating Biodiversity Data into Botanic Collections

**DOI:** 10.3897/BDJ.4.e7971

**Published:** 2016-05-20

**Authors:** Thomas Horn

**Affiliations:** ‡ Molecular Cell Biology, Botanic Institute, Karlsruhe Institute of Technology, Kaiserstraße 2, 76128 Karlsruhe, Germany

**Keywords:** Botanic Collections, Conservation, Invasiveness, Encyclopedia of Life, Catalogue of Life, The Plant List, Biodiversity, Bioinformatics, International Union for Conservation of Nature, Global Invasive Species Information Network, Delivering Alien Invasive Species Inventories for Europe, European Union

## Abstract

**Background:**

Today's species names are entry points into a web of publicly available knowledge and are integral parts of legislation concerning biological conservation and consumer safety. Species information usually is fragmented, can be misleading due to the existence of different names and might even be biased because of an identical name that is used for a different species. Safely navigating through the name space is one of the most challenging tasks when associating names with data and when decisions are made which name to include in legislation. Integrating publicly available dynamic data to characterise plant genetic resources of botanic gardens and other facilities will significantly increase the efficiency of recovering relevant information for research projects, identifying potentially invasive taxa, constructing priority lists and developing DNA-based specimen authentication.

**New information:**

To demonstrate information availability and discuss integration into botanic collections, scientific names derived from botanic gardens were evaluated using the Encyclopedia of Life, The Catalogue of Life and The Plant List. 98.5% of the names could be verified by the combined use of these providers. Comparing taxonomic status information 13 % of the cases were in disagreement. About 7 % of the verified names were found to be included in the International Union for Conservation of Nature Red List, including one extinct taxon and three taxa with the status "extinct in the wild". As second most important factor for biodiversity loss, potential invasiveness was determined. Approximately 4 % of the verified names were detected using the Global Invasive Species Information Network, including 208 invasive taxa. According to Delivering Alien Invasive Species Inventories for Europe around 20 % of the verified names are European alien taxa including 15 of the worst European invasive taxa. Considering alternative names in the data recovery process, success increased up to 18 %.

## Introduction

Before the introduction of binomial nomenclature species names were intended to combine identity and diagnostic description. Starting with Species Plantarum (Linnaeus and Salvius 1753) a species name has been a unique label linked to a (morpho)species supported by a separate diagnostic description. Nowadays the name of a species is not only linked to vast amounts of information, it is also included in legislation concerning biological conservation (e.g. EU council directive 92/43/EEC and EU regulation 1143/2014) and consumer safety (e.g. EU regulation 258/97). Available species information usually is fragmented, can be misleading and might even be biased, because the specimen used for research was not adequately identified. Safely navigating through the name space is one of the most challenging tasks when associating names with data and when decisions are made about which name to include in legislation. The collation and cooperative evaluation of relevant information can lead to a higher level of consistency, prevent information discontinuities and recover those created in the past.

### Names

Species are believed to be natural units of biodiversity and are used in many biological disciplines as empirical units. Research on one of these units, gathering literature or publicly available data, almost always means to consider more than one name (i.e. synonyms, homonyms and spelling variants). To determine the taxonomic status of the species name of interest and to collate all relevant names for research, one can consult taxonomic check lists. These have been setup on a variety of regional levels (i .e . province, state, country) and for particular taxonomic groups (e.g. bird checklist, moss checklist). With the beginning of the new millennium a global checklist for all life forms was established and soon after, a global checklist for plants was setup (see below). The collation of several regional checklists with the aim to build a global checklist helps to reduce ambiguity and provides a single place to search for comprehensive information on the name space of a species.

### Data repositories

In order to collate a uniform and validated index to the world's known species the Integrated Taxonomic Information System (ITIS) and Species 2000 joined forces to set up The Catalogue of Life (COL) in 2001. As of October 2014 it contained 1.5 million accepted and provisionally accepted taxonomic names (341 thousand of Plantae). With a similar agenda but focused on plants the Royal Botanic Gardens Kew and the Missouri Botanical Garden enabled the creation of The Plant List (TPL) combining multiple checklist data sets held by these institutions and other collaborators in 2010. As of September 2013 and the release of version 1.1 TPL contained 1.3 million scientific plant names of which 351 thousand are accepted species names.

With sufficient information on the establishment of the accepted scientific name, including names that are no longer in use, the story of a species unfolds and literature can be screened for additional information. Beyond literature there are freely available scientific data repositories of different kinds. Members of the International Nucleotide Sequence Database Collaboration (INSDC) have been collecting and providing sequence information for 30 years accumulating about 178 million sequences of 340 thousand species and infraspecific epithets. The Barcoding of Life Datasystems (BOLD) supporting the generation and application of DNA barcode data, as of May 2015 offering over 4 million DNA barcode sequences supporting specimen identification. The Global Biodiversity Information Facility (GBIF) provides a single point of access to more than 600 million specimen and occurrence records, shared freely by hundreds of institutions worldwide, making it the biggest biodiversity database on the Internet. Names are also critical when building priority lists, e.g. the Convention on International Trade in Endangered Species of Wild Fauna and Flora (CITES), the International Union for Conservation of Nature (IUCN) or invasive species lists like the Global Invasive Species Information Network (GISIN) and Delivering Alien Invasive Species Inventories for Europe (DAISIE).

The vision of building a species database combining names with all kinds of useful data (Wilson 2003) was realized after E. O. Wilson’s 2007 TED Prize speech by the release of the Encyclopedia of Life (EOL) in February 2008. Connected to scientific names derived from different sources are common names, detailed descriptions, multimedia and many different data subject types (e.g. ecology, geography and molecular biology). For a complete subject list see here.

All of these data providers offer a website where the user can search for information. Some of them also offer the possibility to retrieve information through an application programming interface (API). By using dynamic high-level general-purpose languages (e.g. Perl, Phyton, PHP and others) stakeholders can include relevant data in their own (web) application. Additionally, scientists are able to retrieve and analyse data from many different taxa in little time by using either the API or third party software that facilitates the API (e.g. Banbury and O'Meara 2014).

### Botanic Gardens

Using science as fundamental criteria for the definition of a botanic garden, the first European botanic gardens were established in the mid-16th century [Palmer 1985] and focused solely on the study of medicinal plants. The model of such a "physic garden" became popular and resulted in the reproduction throughout Europe in the following decades. In 1621 the University of Oxford botanic garden (Oxford BG) was the first to be established in the United Kingdom (www.bgci.org/resources/history/).

Today the role of botanic gardens is much more diverse. Support of scientific research and economic endeavors (e.g. Centre of Economic Botany at Royal Botanic Gardens Kew, founded in 1847 [Desmond 1995]), involvement in education, public relations, improvement of human well-being [Waylen 2006] and plant conservation [Ashton 1988, Hurka 1994, Maunder et al. 2001, Donaldson 2009] are some examples.

Botanic Gardens Conservation International (BGCI) is the world authority on botanic gardens and plant conservation. It represents about 700 members, mostly botanic gardens, from 118 countries. A traditional practice among botanic gardens is the exchange of plant (genetic) resources by annually offering seed catalogues from which other gardens can order to develop their own collection. This practice is believed to have started in the late 16 nth century at the Oxford BG [Aplin et al. 2007] and, over the years, has been critically discussed [Havinga et al. 2016, Aplin and Heywood 2008, Heywood 1964, Howard et al. 1964]. A more recent development came with the dawn of the information age. Some gardens now provide access to their botanic collections through a web interface and interested parties can request material for their collection or research online. As a result of the Convention on Biological Diversity (CBD) the International Plant Exchange Network (IPEN) was endorsed by the EU-Consortium and approved by EU-gardens as model to meet the requirements of the CBD on access to genetic resources and benefit-sharing [Robbrecht 2004].

The possibility to develop a collection or scientifically utilize species of botanic collections without the need for expensive expeditions and simultaneously complying to the CBD is appealing. However, the exchange and cultivation of plant species also has less favorable consequences. The introduction of exotic species by botanic gardens has been associated with the potential for escape and evolution of invasiveness [Hulme 2011, Maunder et al. 2001]. The tremendous ecological and economic effects invasive species have [Pimentel et al. 2000] stimulated governments around the world to establish preventive measures (e.g. European Union Regulation No. 1143/2014, United States Federal Law: National Invasive Species Act). Other consequences of cultivation that are of great importance for species conservation with the ultimate goal of reintroduction are a range of threats to the genetic structure of corresponding species [Maunder et al. 2004, Husband and Campbell 2004].

### Aim of the study

The aim of this study was to demonstrate availability and integration of critical information concerning plant genetic resources. As a sample of taxonomic names I used seed catalogues that were received by the botanic garden of the Karlsruhe Institute of Technology in 2014. The assumption that no complete consensus exists about the status of plant taxonomic names was tested and each name was regarded to be potentially associated with additional data. After a list of unique names was compiled, those names were used to retrieve associated data. Exemplarily I used information that is relevant to botanic gardens (i.e. IUCN Red List status and GISIN invasiveness reports). I also tested if the inclusion of alternative names (provided by TPL) would increase information retrieval success. Finally, I discuss the benefits of integrating information in existing management systems of botanic collections.

## Material and methods

Botanic collections (e.g. botanic gardens) contain a vast array of genetic resources that are available for public display, research and education. In the following I describe the use of seed catalogues as a sample of taxon names that are (elsewhere) associated with additional data. I start with the verification of taxon names, evaluate the status of these names and use verified names to retrieve associated information (conservation status, invasiveness and some molecular data). In detail I analysed the content of seed catalogues (i.e. indices seminum, IS) from 134, mostly European, botanic gardens (Fig. 1a), that were electronically available to me at the time. Exemplarily I also included one complete botanic collection (Montoso Gardens, Puerto Rico, USA).

### Data preparation

PDF documents of seed catalogues and text extracts of web sites were converted into XML using pdf2xml converter, parsed to extract taxa names which then were transferred into a local database. The parser was restricted to name patterns of species, subspecies, varieties, forms, cultivars and hybrids of the form "Genus x species". A local database (TPL-DB) was compiled to hold all scientific names and associated information available in CSV format from the TPL website.

### Name evaluation

**Name verification**

To verify the existence of taxon names (Fig. 1b), data from TPL (The Plant List Version 1.1, available from http://www.theplantlist.org/), EOL (Encyclopedia of Life. Available from http://www.eol.org. Accessed March 2015) and COL (Catalogue of Life. Available from http://www.catalogueoflife.org/, Roskov et al. 2014) was considered. EOL and COL provide an application programming interface (API) that I used to access the data (Table 1). TPL on the other hand does not offer an API thus was accessed through TPL-DB.

**Taxonomic status**

In addition to the verification of a name, its taxonomic status was retrieved using TPL and COL. Both sources use two slightly different status terminologies. While COL offers 3 distinct status types ("Accepted name", "Provisionally accepted name" and "Synonym"), TPL offers 4 status types ("Accepted", "Synonym", "Unresolved" and "Misapplied"). To have a unique scheme, status information of COL was adapted to that of TPL by considering "Accepted name" and "Provisionally accepted name" as status type "Accepted". The status "Ambiguous" was introduced by the author as indicator for scientific names with different authorship (homonyms) or very similar spelling variants.

In detail (Fig. 1b), taxonomic status information provided by COL and TPL was retrieved in two steps. First, only exact name matches were used. In case more than one exact match was returned the respective status was set to “Ambiguous”. Secondly, all names that did not return any result (NA) were re-evaluated by retrieving all names related to the next higher hierarchical level (i.e. species in case of infraspecific epithets or genus in case of species names). If any of the returned names was an exact match or very similar (i.e. Levenshtein distance < 3) i t was retained as alternative. In case more than one possible alternative was found the name status was set to “Ambiguous”. If a single alternative match was found the corresponding status returned by the provider was used as result. For subsequent analyses the results of verification and taxonomic name status evaluation were merged and a list of unique names was compiled. In addition to the taxonomic status the source's unique identifier for the taxon was stored.

**Status discrepancies**

To evaluate taxonomic status discrepancies between COL and TPL, the results were combined and the status of each unique name was compared. The status was either identical or not. In the latter case the comparison was saved and used to summarise types of discrepancies.

### Conservation - IUCN Red List status

The list of unique verified taxon names was used to determine the IUCN Red List status for each taxon. First, plant data was downloaded from the IUCN website and installed in a local database. Second, the database was queried and status information was saved. In case a UNL name was not found among the primary names of IUCN, alternative names (i.e. the accepted name and all synonyms of the UNL name) provided by TPL were used to query the database.

### Invasiveness - GISIN and DAISIE

To determine the number and names of potentially invasive taxa cultivated and, more importantly, globally distributed by botanic gardens, I used the GISIN web service to query each unique verified taxon name and retrieve occurrence datasets. Here, I used the same approach as described before, considering alternative names in case the original name did not yield any data. If the dataset contained an entry that reported the taxon as exotic and harmful it was considered to be potentially invasive in its exotic range. To provide for the predominance of European seed catalogues, I compiled a database containing all Magnoliophyta, Gymnospemae, Pteridophyta and Bryophyta taxon names of DAISIE. Again, all unique verified names were checked for occurrence in the local DAISIE database.

### DNA barcodes

The number of public DNA sequence records stored in BOLD for each of the taxon names of the UNL was determined using the BOLD API by firstly determining if the taxon name exists (Taxon Search API) and secondly, if the name was present, taxon data including the number of public sequence records was retrieved (Taxon Data API). Again, in case the original name was not found, I used the alternative name approach as described before.

## Results

Taxonomic names were extracted from name lists of 135 botanic gardens located in 124 European, 6 North American, 4 Asian countries and 1 South American country (Fig. 1a). The resulting database contained 58'990 names, comprising 14'795 unique species, 209 hybrids, 1'147 subspecies, 683 varieties, 85 forms and 1'892 cultivars representing 3'260 genera and 296 families. After excluding all cultivars there were 16'223 names left for validation via EOL, COL and TPL (Suppl. material 2). The assembled TPL name database contained 1'292'889 entries, 99.9 % of Version 1.1 with 1'293'685 name records.

### Name verification

Using exact-search (Fig. 1b), 13'764, 12'939 and 14'338 names could be verified using COL, EOL and TPL respectively, constituting a success rate of 79.8 to 88.4 %. Searching for alternative names (relaxed-search, Fig. 1b) using a Levenshtein distance of < 3 resulted in additional 566, 1'894 and 686 verified names raising the success rate to 88.3 to 92.6 % (Suppl. material 3). 1'894, 1'391 or 1'200 names (7.4 to 11.7 %) could not be verified using COL, EOL and TPL respectively (Fig. 2a). Combining verified names of all three online sources and removing redundant names resulted in 16'224 unique names which were used to retrieve information on conservation status and invasiveness.

### Taxonomic status and discrepancies

Taxonomic name status information is supplied by COL and TPL (Fig. 2b). 66 % of the names verified using TPL are considered to be accepted names, 15 % ambiguous, 12 % synonyms and 6 % unresolved. Using COL, 82 % of taxa names are considered to be accepted, 2.5 % ambiguous and 15 % synonyms.

**Figure 3.**
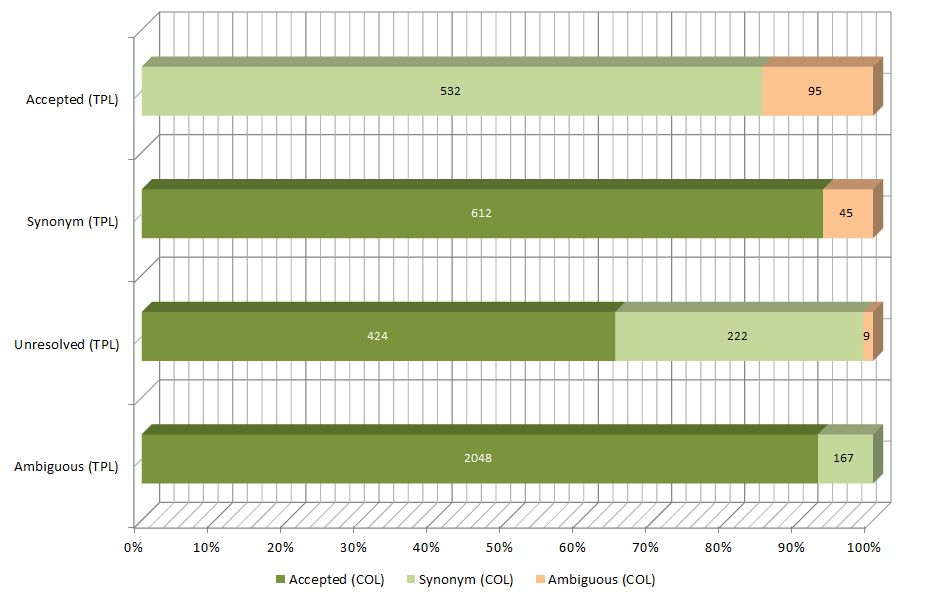
Taxon name status discrepancies between TPL and COL. The figure shows the four status types (accepted, synonym, unresolved and ambiguous) of TPL (y-axis) and the number of cases where a different status (light green = synonym, dark green = accepted and light red = ambiguous) was returned by COL (x-axis). Suppl. material 4

Comparing the status information retrieved during name verification from TPL and COL (Fig. 3) 3'552 discrepancies (23 %) were found (Suppl. material 4). The majority of conflicts (2'048) are based on names accepted by COL but considered ambiguous by using TPL information. Remaining names that COL considered as accepted (1'036) were synonyms (612) or unresolved (424) according to TPL. Names considered to be synonyms by COL (921) were found to be accepted (532), unresolved (222) or ambiguous (167) by TPL. Lastly, a minority of ambiguous COL names (149) were found to be accepted (95), synonyms (45) or unresolved (9) by TPL.

### Conservation - IUCN Red List status

Out of 16'224 unique taxon names (UNL), 1'232 (7.6 %) were found to be assessed by the IUCN Red List (RL). 17.6 % of the names were recovered by including alternative names. One of the taxon names returned the status extinct (EX) - *Cnidoscolus aconitifolius*. According to TPL Cnidoscolus aconitifolius (Mill.) I.M.Johnst. is also known as *Cnidoscolus fragrans* (Kunth) Pohl, which can be found in RL under *C. fragrans* (H.B. K.) Pohl. Three of the taxon names lead to the status extinct in the wild (EW): *Bromus bromoideus*, *Lysimachia minoricensis* and *Mangifera casturi*. 267 (1.7 %) names fell into one of the IUCN Red List threatened categories (vulnerable, endangered and critically endangered) and the remaining 795 included 84 near threatened (27 in the lower risk category), 6 lower risk conservation dependent, 620 least concern (39 on the lower risk category) and 85 data deficient taxon names (Fig. 4a). All taxon names with IUCN Red List status are compiled in Suppl. material 5.

### Invasiveness - GISIN and DAISIE

Of 16'223 unique taxon names 4.3 % (694) have records in the GISIN database. 12.2 % of the names only yielded GISIN records after including alternative names. 650 taxa are report to be exotic, 208 of these are also reported to be harmful (invasive). 39 taxa are neither exotic nor harmful. All exotic and invasive taxon names detected using GISIN are included in Suppl. material 6. DAISIE offers information on 6'091 European alien plant taxa. Evaluating the UNL 19.6 % of the names appear to be European alien taxa. DAISIE also offers a list of the 100 worst invasive aliens in Europe. Among these there are 18 terrestrial plant taxa and 12 of these are found in the UNL. All taxon names detected using DAISIE are included in Suppl. material 7.

### DNA barcodes

Of 16'223 unique taxon names 57.6 % (9'342) can be found in the BOLD taxonomy database. 10.2 % of the names were recovered by including alternative names. Approximately 52 % (8'383) of the names are associated with at least one public barcode record at BOLD. All taxon names and the number of public records are included in Suppl. material 8.

## Discussion

Species - recognizable isolated units of biodiversity - have been described, named, grouped and rearranged for centuries. Information about scientific names and details about the organisms they represent are stored at various places and can be accessed by supplying a specific name that is known by the data provider. The retrieval and allocation of data using a single name or an incomplete set of names frequently is unsuccessful and vital information is missed.

With natural habitats being the primary source for biodiversity samples, botanic gardens, as secondary sources, usually organise their accessions by a single name and do not provide information on synonyms or the status of the name. While data of these institutions is accumulated in BGCI and GBIF, the approach to accumulate taxon specific data on a collection basis, offering additional information and tools of particular interest to managers of such collections, to my knowledge, still has to be implemented. Improving on that part certainly would increase documentation quality and support authenticity of such collections. At the same time the quality of data accumulated by BGCI and GBIF would increase.

Plant conservation offers two examples where "tools" would be of particular interest. What is the current conservational state of a certain taxon or the whole collection? Are any of the taxa known to be invasive, here or elsewhere? In general, to assess a botanic collection, it is very helpful to be able to access various information about organisms represented by scientific names. Searching for specimens with particular attributes, taxonomic or otherwise, it is helpful when information of existing resources (specimens, seeds, DNA, etc.) is also associated with existing information about the organisms of interest. To achieve such a state, it is necessary to work the name space, to incorporate multiple layers for different organisational units (species, communities, habitats) and finally retrieve dynamically updated data from different sources.

### The namespace of EOL, COL and TPL

**Name verification**

Name verification of the current study begins with the binomial of a species for which alternative names (synonyms, homonyms and spelling variants) are returned in the result. Considering the authorship, homonyms can be identified in most cases. Discrepancies in form of different spelling or abbreviation variants can be used to indicate caution before further processing. Comparing verification rates of all three name providers, TPL (92 %), EOL (91 %) and COL (88%) show solid results. Using of Levenshtein distance as a recovery approach is adequate for simple typos. In case of more complex spelling variants, however, a fuzzy algorithm (Rees 2014) would be more appropriate. Most of the 11.6 % of names verified by EOL in the second round (Fig. 2a) of the name analysis are not spelling variants but names that were not found due to the use of the exact search option. This can be explained by an inconsistent name field format. In most of the cases the field only contains the scientific name, like in the case of COL and TPL. In the mentioned instances, however, the field also contains the authority and makes an exact search, not using the authority, impossible.

While all data providers used in this study are capable to direct a request, using a synonym, to the currently accepted name, other, usually less global data providers use a limited name space. Requests made with a synonym not included by the provider will subsequently fail to return information. The IUCN red list (as of 2014), although rather global, for example, only has limited information on synonyms available. By integrating all known names into a request, chances for successfully retrieving information will be maximized (see Fig. 4a and discussion below). In cases the data of interest has already been collected and associated with the right name(s), one can access it more easily (e.g. EOL).

**Taxonomic status and discrepancies**

Only COL and TPL directly provide status information with the name search result. According to TPL 98 % of all status values were directly derived from the data source that supplied the name record while only 2 % are a result of automated conflict resolution processes. According to COL their data is based on global species databases that have been validated for inclusion by independent peer reviews.

The number of name status conflicts found between COL and TPL (23 %) appears very high. However, more than half of these cases are ambiguities detected using TPL information (i.e. homonyms) and thus are not truly conflicts of taxonomic status. Aside from an unknown number of conflicting cases due to homonyms, 12 % of the evaluated names are associated with different taxonomic status. Assuming a similar proportion for the cases of homonyms while including the authority of the name, the total number of conflicts would increase by approximately 2 %. The main fraction (66 %) of these conflicts are cases where one source considers the name as accepted and the other as synonym. The remaining conflicts (34 %) are based on a TPL specific status, which highlights currently unresolved names. Considering the provided status information, TPL apparently offers a more heterogeneous view. As mentioned above, a much higher degree of name ambiguity can be detected using TPL. About 4 - 6 times more names are considered ambiguous because of the existence of homonyms, many of which are not found using COL. Besides the mentioned additional status (i.e. unresolved), confidence of the declared status is indicated for each name by a three star system (high, moderate and low confidence). All these features and the high name verification rate makes TPL a distinguished source for plant names.

The primary concern of this study was to verify names and find associated information. Having an exhaustive name space increases the number of potential hits when mining for data (see Fig. 4). Complete and accurate information on a name means being able to take all information into consideration when evaluating associated data of interest.

**The extended supply of information provided by EOL**

Along with a scientific name, synonyms and common names in different languages, EOL provides a place where a plenitude of other information on species can be found. Only recently EOL's TraitBank (Parr et al. 2015, Parr et al. 2014) was introduced. It is, in part, a text mining approach that infers traits (e.g. distribution, habitat, elevation, interactions, etc.) from the occurrence of specific patterns (Ontology of Biological Attributes, Dietze et al. 2014) in content associated with a species and further enriches the web of species knowledge. While in the past access to relevant information was restricted and manual search was laborious, today, thanks to the effort of EOL and others that prepare and provide data on a freely accessible platform, this has changed considerably. Now we can answer questions more quickly and approach them in new ways (Hart 2014, Wright and Seltmann 2014, Barve 2014).

### Genetic resources of botanic gardens

Botanic gardens cultivate and store many different plant species for public display, education and scientific research. While exact species delimitation and taxonomic placement appears to be of less importance to the public and only partially to education, it is of utmost importance to scientific research.

**Consequences of ex-situ cultivation and the quality of current botanic garden collections**

Botanic gardens are, by their very nature, places where many different species, which under normal circumstances would never meet, are being brought together (artificial sympatry). Additionally, the cultivation of these species inevitably means that they are put into a novel environment (artificial selection). Both factors entail complex consequences that are relevant for our understanding of plant evolution and for conservation biological projects (Ashton 1988, Husband and Campbell 2004, Maunder et al. 2004, Schaal and Leverich 2004, Donaldson 2009). To study related phenomena, information regarding the origin and distribution of botanic resources are vital.

Judging from the analysed seed lists there are still many botanic collections where information on the origin of specimens is missing or has been lost (data not shown), a fact that reduces scientific value of these collections considerably (Böcher and Hjerting 1964). The effects of cultivation (e.g. artificial selection, hybridization, genetic bottlenecks) (Barrett et al. 1991, Maunder et al. 2004) cannot be traced. Furthermore, results obtained from such a specimen may not be feasible to draw generalisation for the species, since it is not clear if this organism, in regard of the particular characteristic, is still a common representative of the species (Maunder et al. 2001, Sharrock and Jones 2011). For that, a new adequate (Brown and Briggs 1991) sample would be necessary. To increase the scientific value of botanic collections at least those accessions where a replacement can be achieved with minimal effort (i.e. annuals and perennial herbs) should be replaced with adequately documented ones.

Since a botanic garden will, in most cases, be artificial, and open pollination is expected to be the common reproductive mode, the primary objective maintaining a botanic garden in a scientific sense is sufficient documentation and verification of authenticity of specimens. With access to relevant data (e.g. checklists, determination keys and taxonomic experts) this task becomes much easier. By dynamically incorporating data from external sources into a collection management system, local staff and scientists alike have access to specimen related information that can be used collectively to achieve this vital objective.

As demonstrated in this study for most names (86 %) TPL and COL are in agreement on the taxonomic status. For cases of disagreement the more specialized source (i.e. TPL) should be consulted. Additionally, if the question which other plants might be confused with the one of interest is of particular interest (e.g. toxicology, food diagnostics) TPL offers the most complete dataset in terms of synonyms and homonyms and also includes status information (i.e. unresolved) that indicates the need for further taxonomic study.

**Authenticity of genetic resources**

The author has personally worked with specimens from several plant genera obtained from different botanic gardens and found that misidentifications appear to be as common as indicated elsewhere (Carolin 1957, Hurka 1994) and represent another problem for education and research. To keep up with taxonomic research the integration of data from external sources is very helpful. But how does this help maintaining an authentic collection ?

Having access to determination keys is one thing, but to apply this information to identify specimens to species requires experience. Different sources (including EOL) provide such expertise in form of images from herbaria and close-ups of diagnostic traits from living specimens. This solution, however, is still a work in progress. Such interactive keys are mostly regionally or taxonomically restricted and thus are not yet a universally applicable approach.

A relatively new approach of specimen identification, addressing the declining numbers of taxonomic experts, is DNA barcoding (sensu Hebert et al. 2003). It uses a small standardized region of the (plant) genome to determine the species name of a specimen. By using the information of the DNA, this approach is not limited to a certain developmental stage and is not biased by environmental factors. However, it has been shown to be of very limited use for species-level specimen identification in land plants when using the officially proposed (CBOL 2009) chloroplast markers (rbcL and matK). Identification success rates increase when using more variable marker regions (Federici et al. 2013, Roy et al. 2010, Seberg and Petersen 2009, Taberlet et al. 2007). Most markers, however, have been shown to be unable to resolve closely related taxa as single barcode marker. Additionally, to apply DNA barcoding for specimen identification, a database containing DNA barcodes of species of interest is necessary. In the current study at least one DNA barcode sequence exists at BOLD for approximately 58 % of the analysed taxa. It is therefore reasonable to approach specimen identification using DNA barcoding with different priorities (i.e. species recovery rate over marker universality) and at the same time to keep an open mind about alternative approaches.

The development of sequence markers based on conventional DNA fingerprinting methods (e.g. RAPD sensu Williams et al. 1990, SRAP sensu Li and Quiros 2001) is too laborious to be practical. Using highly species specific functional DNA fingerprinting patterns (Bardini et al. 2004, Breviario et al. 2007, Poczai et al. 2013) universally obtained by fast and reliable methods (Gavazzi et al. 2012), however, might be a feasible alternative. One particular advantage of this approach is its capability of detecting and differentiating hybrids [Braglia et al. 2014] which, due to open pollination, are expected to occur more frequently in botanic gardens. Something which is impossible to achieve with plastid based DNA barcoding markers.

### Conservation of nature

**Threatened taxa**

In a previous analysis of the IUCN dataset *E. minima* Jacq. ex DC., collected at 2500 m height in Col du Galibier (France), offered in the IS of BGU Lautaret 2014, was detected. Additionally *Euphrasia minima* Schleich. was detected in the IS of CJB Geneva. According to IUCN the taxon is a synonym for *Euphrasia mendoncae* Samp., which is extinct. However, the name *E. minima* Jacq. ex DC. is not a synonym for *Euphrasia mendoncae* Samp. (A. Gröger personal communication). In the most recent analysis alternative names were considered before checking IUCN synonyms and another species name was detected that is considered to be extinct by IUCN. *Cnidoscolus fragrans* (Kunth) Pohl, according to TPL a synonym for *Cnidoscolus aconitifolius* (Mill.) I.M.Johnst. is found in the botanic collection of the Montoso botanic garden, Puerto Rico. Both cases demonstrate the importance of names. Firstly the correct use of alternative names and secondly the consideration of alternative names during information retrieval.

Sharrock and Jones (2011) identified 42 % (808) of European threatened taxa in 285 European botanic garden collections using data from Botanic Gardens Conservation International. In the current study 267 taxa of IUCN threatened categories were detected in botanic gardens, representing approximately 14 % of European threatened taxa sensu Sharrock and Jones (2011). With 44 % of the number of gardens considered by Sharrock and Jones the proportion detected in the current study appears to be quite low. However, the current study used mostly seed catalogues which usually represent only a fraction of a garden's collection. While this is sufficient to demonstrate data associations of names, it might be an inadequate sample to draw conclusions on conservation efforts of involved gardens. Additionally, conservation efforts are not simply expressed by the quantity of threatened taxa maintained or distributed by a botanic garden (Havens et al. 2006). Conservation efforts start with promoting awareness (Williams et al. 2015), awareness of the impact of human populations on ecosystems and the intrinsic value of living communities. Public opinion, education and awareness are key aspects for the establishment of an evolutionary ethic as part of our societies which would lead to a broad public recognition that the existence of other species is an integral part of our own (Hurka 1994, Frankel 1974).

**Invasive taxa**

On the 1st of January 2015 an EU regulation on the prevention and management of the introduction and spread of invasive alien species (No. 1143/2014) came into force. It aims to address the adverse impact alien invasive species have on biodiversity, ecosystem services, human health and the economy in the EU member states. Botanic gardens, without doubt, create artificial situations for species. Hybridization, as one possible consequence, has been shown to be an important factor in evolution (Martinsen and Whitham 1994, Whitham et al. 1994) and as a promoter of invasiveness (Ellstrand and Schierenbeck 2000). For the European Botanic Garden Consortium (EBGC) "it is vital that Botanic Gardens take steps to prevent future problem taxa from establishing through their collections" and direct stakeholders to "Initiatives such as Delivering Alien Invasive Species Inventories for Europe and North European and Baltic Network on Invasive Species as well as National Initiatives, such as Harmonia - Invasive species in Belgium " which provide detailed databases on respective species. One major problem mentioned is, that "it can be difficult for garden managers and curators to obtain summary lists that provide at a glance indications of problem taxa". This difficulty subsequently leads to seed lists containing potentially invasive species (e.g. *Acacia dealbata* Link, *Ailanthus altissima* (Mill.) Swingle, *Echinocystis lobata* (Michx.) Torr. & Gray., *Rhododendron ponticum* L.) without any note or indication. The identification of high-risk taxa is the responsibility of both, distributing and receiving parties, may that be botanic gardens or the horticultural industry (Baskin 2002, Wittenberg and Cock 2004). I demonstrated the integration of data from publicly available sources (GISIN and DAISIE) to provide at a glance indications of potentially invasive taxa. If generally applied, garden managers and scientists would be able to act with little latency evaluating and eliminating potential threats (Hulme 2011, Hulme 2014, Sharrock 2011). For this to happen data providers need to establish an API and collection managers or stakeholders should discuss the creation of an administrative solution suitable for botanic gardens. Since many data providers already offer an API the most likely obstacle in implementing such a tool is the transition of collection data from the "old" to a "new" system. Aside from an integrated version there is always the possibility of a "light" version: a list matching service similar to that offered by COL for taxonomic name status (http://www.catalogueoflife.org/content/list-matching-service). Instead of the name status, information on invasiveness would be returned for all supplied names.

### Conclusion

In this study I used scientific names sampled from botanic collections (mostly seed catalogues). I started with the verification of taxon names, evaluated the status of these names and used verified names to retrieve associated information. For that I chose the topics conservation and invasiveness because both appear to be relevant for todays botanic gardens. Many other types of information associated with taxonomic names can be retrieved and used for science, education and the development of botanic collections. Botanic gardens represent one of the major sources of plant genetic resources which is why quality of these resources is of paramount importance. The quality of these resources is reflected by authenticity and sufficient documentation. Authenticity in the past had been secured by one or more specialists - curators. Today the number of these specialists is declining and other methods of authentication need to be considered. A rich documentation of plant genetic resources consists of specimen specific information (e.g. origin of the specimen) and taxon specific information (e.g. associated names, natural habitat, distribution, etc.). The aim of this study was to demonstrate the integration of taxon specific information into botanic collections. Information that can be used by collection managers to assess potential invasiveness in the blink of an eye and by scientists for example to easily find all red flowered plants. Information that is either static or frequently updated (dynamic) by data providers. For the location and retrieval of such information the taxonomic name plays a central role. TPL offers the most promising dataset of plant names with high name coverage and extended information on the taxonomic status. To verify the identity of a specimen the accepted name will lead to the diagnostic description. With a verified specimen, studies can commence and, for example, DNA based authentication can be established. The accepted name leads to alternative names that can lead to additional information, like literature using an old name. With increasing levels of publicly available data through portals like EOL and publishers supporting open data sharing (e.g. Smith et al. 2013, Kenall et al. 2014), a dynamic integration of data will ultimately revolutionize the view on genetic resources and how ecological and evolutionary science is approached.

## Supplementary Material

Supplementary material 1 List of botanic gardens Data type: CSV
Brief description: List of botanic gardens of which seed lists were used in this study
File: oo_74709.csv Thomas Horn

Supplementary material 2 Unique Taxon Name List Data type: CSV
Brief description: List of unique taxon names (excluding cultivars) retrieved from indices seminum used in this study.
File: oo_80358.csv Thomas Horn

Supplementary material 3 Verified names and taxonomic status provided by COL, EOL and TPL Data type: CSV
Brief description: List of all names verified by COL, EOL and TPL including source id and taxonomic status. EOL does not return a taxonomic status with a search result. The column taxonomic status is therefore used to indicate if the name was found in the "title" or "content" field of the result.
File: oo_74360.csv Thomas Horn

Supplementary material 4 Taxonomic name status - discrepancies between COL and TPL Data type: CSV
Brief description: All cases where the taxonomic name status was different between the two sources (TPL and COL) are contained in this list.
File: oo_74356.csv Thomas Horn

Supplementary material 5 Taxa with IUCN Redlist status Data type: CSV
Brief description: All 1232 taxon names with IUCN Redlist status. Extinct (EX), Extinct in the wild (EW), Vulnerable (VU), Endangered (EN), critically Endangered (CR), Lower Risk (LR): Near threatened (NT,nt), Least Concern (LC,lc), Conservation dependent (cd) and Data deficient (DD). Column "Source" indicates if the original name was found in the Redlist (RL), if the original name was found as synonym in the Redlist (RLsynonym) or if an alternative name from TPL (TPLsynonym or TPLaccepted) was found in the Redlist.
File: oo_80748.csv Thomas Horn

Supplementary material 6 Taxa found at GISIN Data type: CSV
Brief description: All 694 taxon names found in GISIN and their status. The column "type" indicates if the original name yielded the status information (original) or if an alternative name provided by TPL yielded the status information (Accepted, Synonym and Unresolved).
File: oo_80750.csv Thomas Horn

Supplementary material 7 DAISIE exotic and invasive taxa Data type: CSV
Brief description: All exotic and invasive taxa detected using DAISIE
File: oo_74926.csv Thomas Horn

Supplementary material 8 Taxa with BOLD public records Data type: CSV
Brief description: List of 8383 taxon names with the number of public records in BOLD. The column "type" indicates if the original name was found at BOLD or if an alternative name from TPL (TPLsynonym or TPLaccepted) was found at BOLD.
File: oo_80746.csv Thomas Horn

## Figures and Tables

**Figure 1a.**
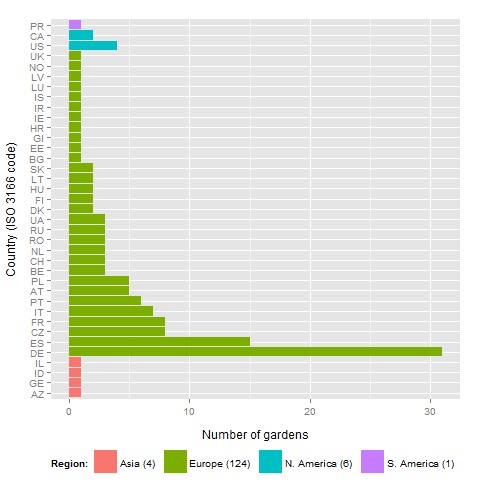
Origin of the 135 botanic gardens from whose seed catalogues taxon names were extracted.

**Figure 1b.**
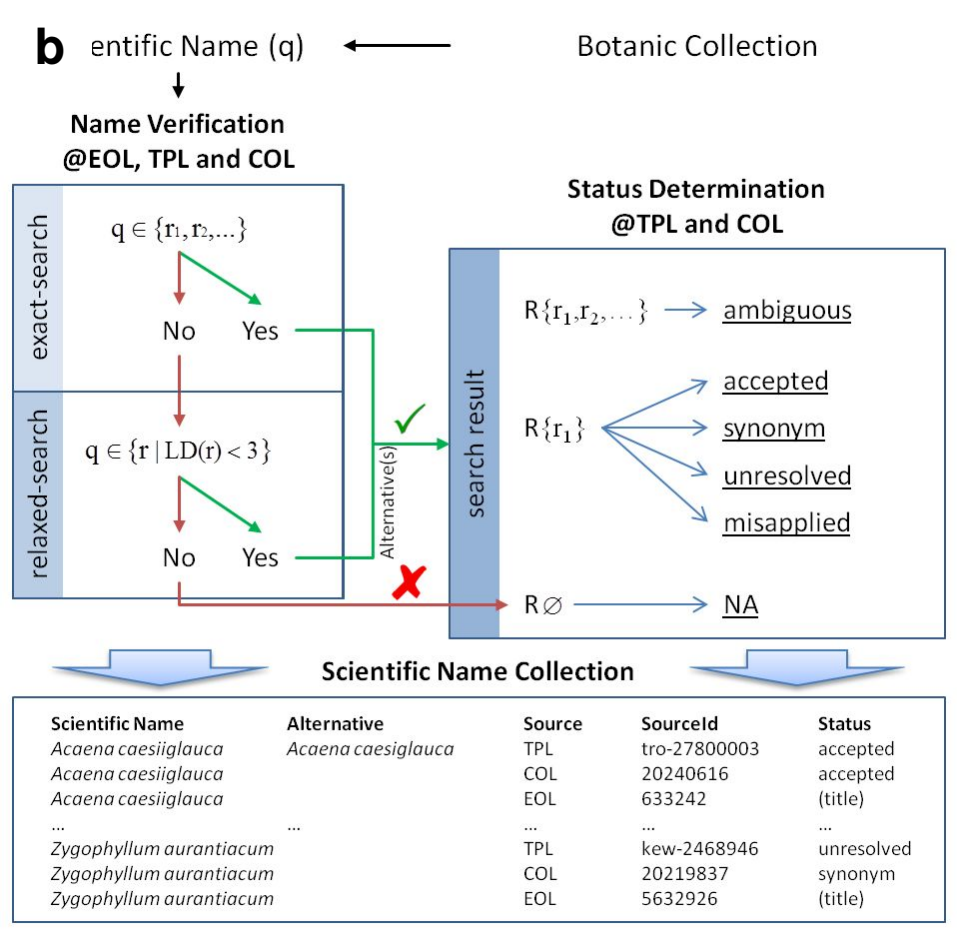
Schematic representation of the name verification and taxonomic status determination process. The existence of a particular name in one of the databases (@EOL, TPL and COL) is considered as verification. After a name is found or alternative names (LD = Levenshtein Distance) are available, the taxonomic status is determined by considering the number of times a name was found (ambiguous if more than one result was returned) and the status returned by the database. The result is saved in a scientific name collection which is used to evaluate taxonomic status discrepancies and to compile a unique name list for further information retrieval (i.e. conservation state @IUCN Red List and invasiveness reports @GISIN and @DAISIE).

**Figure 2a.**
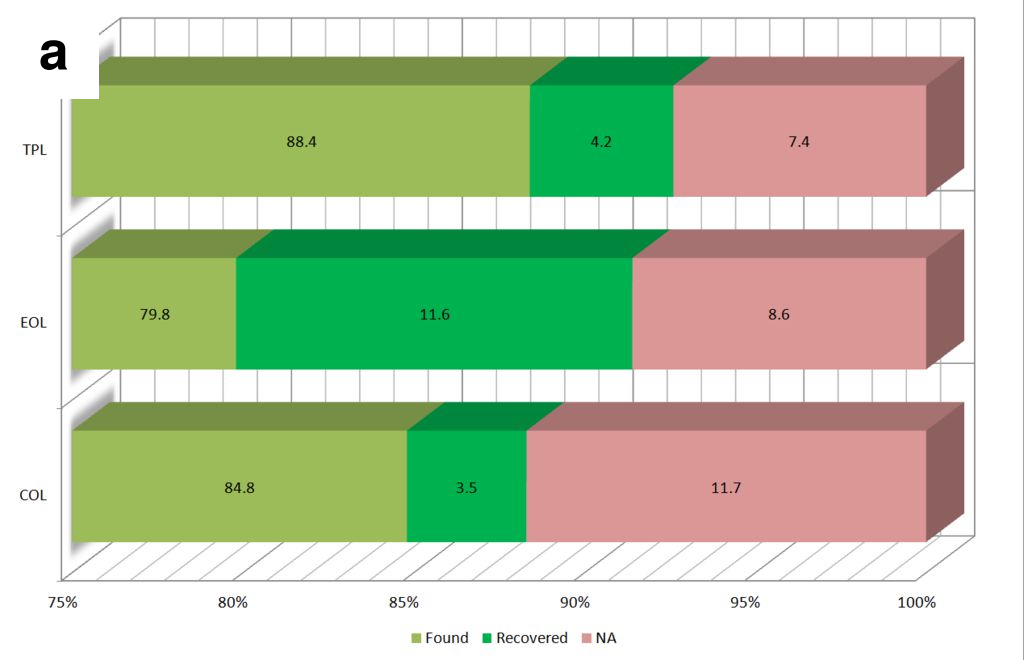
Overview on the verification of taxonomic names and the recovery by using Levenshtein distance < 3 using data from three online sources (TPL, EOL and COL).

**Figure 2b.**
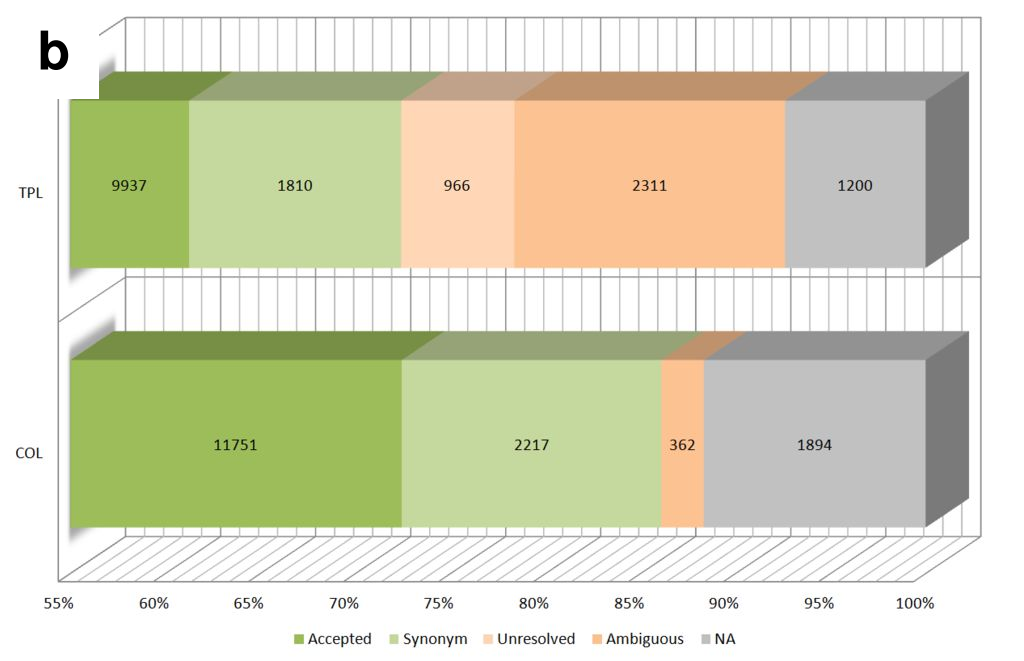
Overview on the taxonomic name status (accepted, synonym, unresolved, ambiguous and NA = not available) of 16'224 unique taxon names determined using two online sources (TPL and COL).

**Figure 4a.**
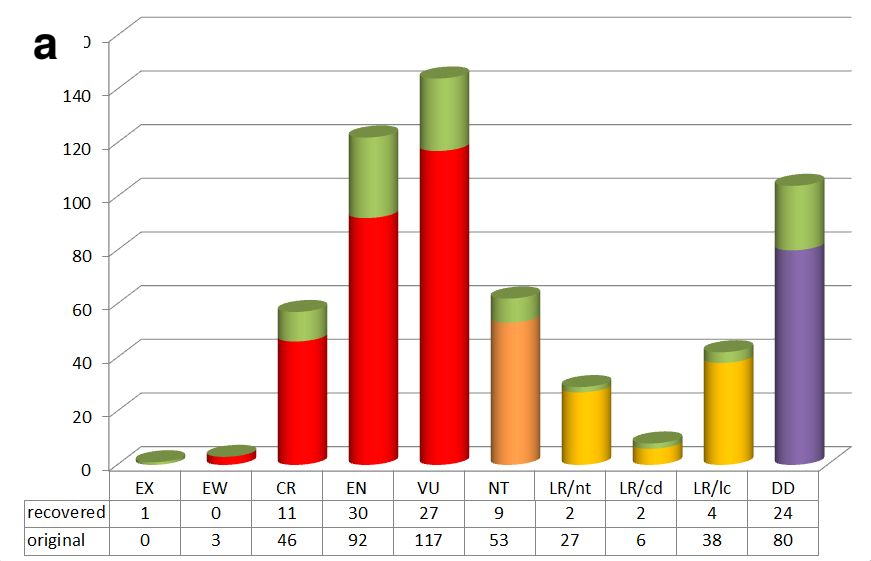
IUCN Red List status of 572 taxon names. The proportion of recovered names after considering alternative names provided by TPL is indicated in light green. Status categories include Extinct: Extinct (EX) and Extinct in the wild (EW); Threatened: Vulnerable (VU), Endangered (EN) and critically Endangered (CR); Others and Lower Risk (LR, yellow): Near threatened (NT, nt, orange), Least Concern (LC, lc), Conservation dependent (cd); and Data deficient (DD, violet). Taxon names (660, including 107 recovered names) with the status LC have been excluded from the graph.

**Figure 4b.**
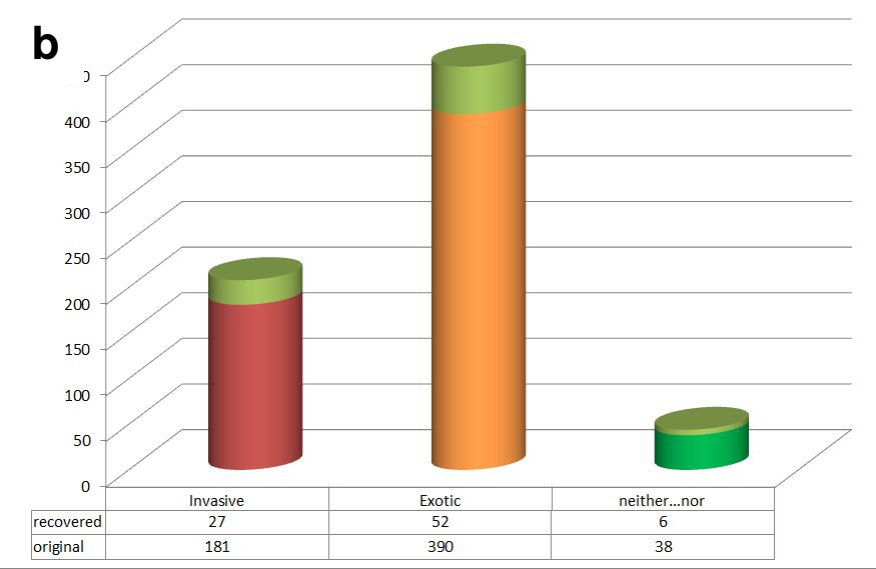
GISIN status of 694 taxon names. The proportion of recovered names after considering alternative names provided by TPL is indicated in light green. Status categories shown are Invasive (recorded as exotic and harmful), Exotic and neither exotic nor harmful.

**Table 1.** API functions and data output format of data sources.

**Source**	**API function**	**Output format**
EOL	eol.org/api/search/1.0.json	JSON
TPL	local mysql database	SQL
COL	www.catalogueoflife.org/webservices/status/query/	XML
